# A Survey of Allergic Consumers and Allergists on Precautionary Allergen Labelling: Where Do We Go from Here?

**DOI:** 10.3390/nu17091556

**Published:** 2025-04-30

**Authors:** François Graham, Susan Waserman, Jennifer Gerdts, Beatrice Povolo, Yvette Bonvalot, Sébastien La Vieille

**Affiliations:** 1 Division of Allergy and Clinical Immunology, Department of Medicine, Centre Hospitalier de l’Université de Montréal (CHUM), Montréal, QC H2X 0C1, Canada; 2 Department of Allergy and Immunology, Centre Hospitalier Universitaire Sainte-Justine, Montréal, QC H3T 1C5, Canada; 3 Division of Clinical Immunology and Allergy, Department of Medicine, McMaster University, Hamilton, ON L8S 4L8, Canada; waserman@mcmaster.ca; 4 Food Allergy Canada, Toronto, ON M2J 4V8, Canada; jgerdts@foodallergycanada.ca (J.G.); bpovolo@foodallergycanada.ca (B.P.); 5 Food Directorate, Health Canada, Ottawa, ON K1A 0K9, Canada; yvette.bonvalot@hc-sc.gc.ca (Y.B.); sebastien.lavieille@hc-sc.gc.ca (S.L.V.); 6 Département de Santé Environnementale et de Santé au Travail, École de Santé Publique de l’Université de Montréal, Montréal, QC H3N 1X9, Canada; 7 Department of Food Sciences, Université Laval, Québec City, QC G1V 0A6, Canada

**Keywords:** food allergy, oral food challenge, eliciting dose, reference dose, threshold, precautionary allergen labelling

## Abstract

Background: Despite the widespread use of precautionary allergen labelling (PAL) by manufacturers, PAL is not always used consistently and can be a source of misinterpretation by consumers and allergists. Although its use is not specifically regulated in Canada, some voluntary guidelines exist. The aims of this study were to investigate allergic consumers’ and clinicians’ understanding of PAL, to describe consumers’ attitudes towards products with PAL, and to examine recommendations given by clinicians to their patients about these products. We also compared two groups of consumers enrolled in this study, since the majority of them (72%) were registered in the Food Allergy Canada database and the others (28%) came from representative consumers of the general population. Methods: An online survey was sent from 2 to 28 December 2021 to allergic consumers registered with Food Allergy Canada’s database and to a group of allergic consumers extracted from a panel representative of the general population and not registered with Food Allergy Canada (third-party panel). All consumer participants had a food allergy or were a parent/caregiver of a child with food allergy and had to be diagnosed by a medical professional. Considering that consumers registered via the Food Allergy Canada database could be more informed about labelling than the third-party consumer panel, we conducted a multivariate analysis (logistic regression) on the key variables related to PAL allowing to compare these two groups of participants. In addition, a separate online survey was sent to allergist members of the Canadian Society of Allergy and Clinical Immunology and provincial associations to investigate their understanding of PAL from 12 November 2021 to 16 January 2022. Results: A total of 1080 consumers and 63 allergists (29% of allergists in Canada) responded to the surveys. Fifty percent of consumers were adults with food allergy, and 50% were a parent/caregiver of a child with food allergy. Food allergy was diagnosed most commonly by an allergist in 76% of the cases. Fifty-four percent of consumers purchased products with a PAL statement at least occasionally, and more than half of consumers (53%) considered PAL a very useful tool. Most surveyed individuals (59%) had not heard of the term “individual allergen threshold” or had heard the term but did not know what it meant. The same allergic consumers were reluctant to buy food products with even a small amount of their allergen (i.e., a dose that would not trigger an allergic reaction in the vast majority of them). Half of allergists reported PAL was not useful in its current form, and 83% supported the consumption of foods with PAL to their patients in some circumstances. Conclusion: While most consumers are somewhat confident in the accuracy of ingredient information on pre-packaged foods, interpretation of PAL remains confusing by many allergic consumers. If changes are to be made based on allergen thresholds, a multi-stakeholder approach will be required with greater consumer and allergist education on risk assessment concepts to facilitate the implementation of allergen population thresholds into the application of PAL.

## 1. Background

The prevalence of food allergy (FA) based on history and/or physician diagnosis is estimated at 6.1% in Canada [1], and FA is one of the primary causes of emergency room visits for anaphylaxis [2]. The management of immunoglobulin E (IgE)-mediated FA generally involves strict avoidance combined with treatment of accidental ingestions. In addition to the potential risk of fatal anaphylaxis, FA is associated with a considerable impact on quality of life (QoL) for patients and their families [3,4]. A major source of stress for patients living with food allergies is the fear of reacting to small amounts of allergens, such as in processed foods with precautionary allergen labelling (PAL) [5].

Health Canada is the federal department responsible for (i) food safety policies and standards for allergen labelling, (ii) health risk assessments in support of investigations and (iii) information for Canadians about potential health risks. Priority food allergens determined by Health Canada include peanut, tree nuts, soy, wheat and triticale, egg, milk, fish, crustaceans and molluscs, sesame, and mustard [6]. According to food labelling regulations, the common name of the priority food allergens, gluten sources (wheat, triticale, barley, rye, oats) and added sulphites must also be included on food labels. A PAL, i.e., a statement such as “may contain”, “may contain traces”, or “manufactured in the same facility”, to name a few, is a declaration on the label of a pre-packaged food of the possible inadvertent presence of a priority allergen. These statements are typically applied by the food industry when allergens are not intentionally added as ingredients but may be present due to shared equipment or supply chain factors. Because some of these statements may not accurately reflect the hazard, Health Canada and the Canadian Food Inspection Agency (CFIA) recommend that food manufacturers and importers use only one PAL statement on food labels, “May contain X”, where X is the name by which the allergen is commonly known [7].

The use of PAL is the responsibility of food manufacturers and importers and is not specifically regulated in Canada and in most other countries, leading to uneven application and consumer confusion [8,9,10]. Moreover, the variety of detection methods and analytical cut-offs used to measure allergen amounts further complicates comparability of PAL practices internationally.

However, in Canada, if the pre-packaged food is produced in a way that poses a risk to allergic consumers due to cross-contact, then the manufacturer has to address this risk, either through PAL or by changing the way the food is manufactured. In addition, under Section 5.1 of the Canadian Food and Drugs Act, any precautionary labelling statements cannot be untruthful or misleading [11]. Lastly, PAL should only be used by manufacturers when, despite all reasonable measures including good manufacturing practices (GMPs), the inadvertent presence of allergens in food is unavoidable.

Unfortunately, despite these food labelling regulations, there is no standardized, consistent approach to applying PAL. In the absence of a risk-based approach, many products with PAL do not contain food allergens, while others without PAL have been found to contain allergen concentrations that are likely to trigger an allergic reaction in a significant proportion of the at-risk population. This situation presents a challenge for consumers with food allergies [12,13], all the more so given recent shifts in global supply chains, which might increase the risk of unintended botanical impurities in food ingredients—adding another layer of complexity to allergen traceability and consumer protection [14].

Current discussions by food manufacturers, scientists and international organizations such as the Food and Agriculture Organization/World Health Organization (FAO/WHO) on the use of PAL involve a risk-based approach for allergen management, including the use of population thresholds (also called population eliciting doses or EDs) [13,15,16,17,18]. The objective of these discussions is to help the food industry determine the concentration of unintended allergen that represents a health hazard so as to inform when to apply PAL for certain priority allergens. One strategy involves PAL recommendations based on population EDs predicted to trigger an allergic reaction in 1% or 5% of allergic subjects (the ED_01_ and ED_05_, respectively) [18,19]. The theoretical risk of anaphylaxis at these doses in the population with FA is very low (2.3 episodes of anaphylactic events per 1000 exposures in the peanut-allergic population at the ED_05_ level) [20,21], although whether this risk is acceptable or not to most consumers remains debatable [22]. With this strategy, no PAL would be used if the amount of allergen in the food is kept below the chosen threshold (ED_01_ or ED_05_), and this could ultimately help reduce the amount of packaged foods with PAL.

The aims of this study were to investigate allergic consumers and clinicians’ understanding of PAL, to describe consumer’s attitudes on products with PAL and to examine recommendations given by clinicians to their patients about these products. Subjects were also surveyed on the use of allergen thresholds as an allergen risk management strategy to better inform PAL.

## 2. Methods

### 2.1. Data Collection

***Consumer survey***: an online survey on PAL was conducted from 2–28 December 2021. The surveyed targeted two distinct and independent sub-groups: one consisting of individuals from Food Allergy Canada (FAC)’s database (Canadian non-profit organization dedicated to food allergic individuals) and the other comprising allergic consumers from a third-party panel that is representative of the general population but not registered with FAC (Supplementary Materials File S1). Participants were required to be 18 years or older with a confirmed food allergy (FA), or parents/caregivers of a child with FA. Those who qualified as both an adult with FA and a parent/caregiver of a child with FA were asked to complete the survey from the perspective of an adult with a FA. All FA diagnoses were confirmed by a medical professional (paediatrician, family physician, allergist, emergency department physician, or gastroenterologist). Although initial self-diagnosis was reported by some participants (*n* = 135), all had their diagnosis subsequently confirmed by a clinician. However, all data were collected online and are self-reported. Participation was voluntary, and anonymized answers were collected for statistical analysis.

Ethics board exemption for this study was granted by the CHU Sainte-Justine’s research ethics board as an activity that did not meet the criteria for research requiring review outlined in article 2.1 of the Tri-Council Policy Statement: Ethical Conduct for Research Involving Humans (TCPS 2) of Canada [23].

***Allergist survey***: Another online survey (SurveyMonkey^®^, San Mateo, CA, USA) was sent to members of the Canadian Society of Allergy and Clinical Immunology (CSACI) as well as Ontario, Quebec, and British Columbia provincial allergist associations from 12 November 2021, to 16 January 2022. All members were allergists and clinical immunologists practicing in Canada. The survey consisted of 16 questions (Supplementary Materials File S2). Ethics approval was obtained from the Hamilton Integrated Research Ethics Board of McMaster University (Project number 13589).

### 2.2. Statistical Analysis

FAC and PANEL sub-samples were independent, and participants were randomly selected. To ensure the representativeness of our two consumer survey samples, thereby enhancing the generalizability and accuracy of our conclusions, a ranking technique for weighting participants was employed. Survey participants were weighted based on two key variables: their food allergy (FA) category (adult with FA or parent of a child with FA) and their region of residence within Canada, categorized into five main regions (Atlantic, Quebec, Ontario, Prairies, and British Columbia). Additionally, a constraint was imposed to achieve a representative sample of 540 participants in each FA category.

Descriptive and inferential statistics (Table 1, Table 2, Table 3, Table 4 and Table 5), including weighted counts and percentage comparisons between the two groups of the consumer survey (FAC Database vs. 3rd party panel) were generated using the R “stats” package version 4.4.3. The “prop.test” function was employed to conduct two-tailed z-tests. The null hypothesis was that FAC Database results were equivalent to those from the 3rd party panel, with the alternative hypothesis being that FAC Database results differed from those of the 3rd party panel. Before employing Z-tests to assess the differences in proportions, we verified the assumptions required for their valid application. These assumptions included ensuring that the expected number of successes and failures in each sample group were at least 5, a prerequisite for the reliable approximation of the binomial distribution to the normal distribution, as well as sample independence and randomness. *p*-values were adjusted for multiple comparisons using the Benjamini-Hochberg procedure to control the false discovery rate (FDR) (refer to Table 1, Table 3 and Table 5). The Benjamini–Hochberg procedure is a common and recommended practice in statistical analyses involving multiple tests to reduce the risk of Type I errors, which are false positives that occur purely by chance.

Both univariate and multivariate weighted logistic regression analyses were performed using the R ‘glm’ package. Similar to Z-tests, *p*-values for both univariate and multivariate weighted logistic regressions were adjusted for each outcome using the Benjamini–Hochberg procedure to control the false discovery rate (FDR) (refer to Table 2 and Table 4).

For all inferential testing, we maintained a significance level of 0.05, considering results statistically significant at *p*-values less than 0.05. Prior to conducting multivariate logistic regression analyses, we thoroughly assessed key model prerequisites to ensure robust performance. These included verifying adequate sample size using the events-per-variable (EPV) rule—ensuring a minimum of 10 to 30 events per predictor variable—checking for the absence of complete separation by reviewing extreme odds ratios and confidence intervals, assessing multicollinearity through Variance Inflation Factor (VIF) values, and evaluating model fit using pseudo R-squared and concordance (C) statistics.

A single instance of potential multicollinearity was identified between the age variable (originally categorized as <35, 35–54, and >54) and the FA group (parents vs. adults). To address this, we excluded the FA group and re-categorized age into four groups: <18, 18–34, 35–54, and >54. All other prerequisite checks were satisfactorily met.

## 3. Results

### 3.1. Consumer Survey

A total of 1080 questionnaires were completed for the consumer survey sample, including 826 from FAC’s database and 254 from a third-party panel. The unweighted sample composition was 453 adults with Food Allergy (FA) (42%) and 627 parents of a child with FA (58%). To achieve balanced representation, the final weighted sample was adjusted to comprise an equal distribution: 50% adults with FA and 50% parents of a child with FA. This weighting adjustment resulted in representing 774 participants from FAC’s database and 306 from the third-party panel in the weighted sample. All tables and results presented in the subsequent sections of this paper are based on these weighted results. Characteristics of surveyed participants are detailed in Table 1.

***Food labels***: The majority of the sampled consumers read food ingredient labels all the time (58%). Reading labels was significantly more common among FAC members (68%) and for those younger than 54 years old. However, there was no significant difference between those who had been prescribed epinephrine, patients who received a food allergy diagnosis by an allergist as opposed to another health care professional, or patients with multiple food allergies compared to those with a single food allergy. Finally, gender and household income were not criteria that influenced the decision to read ingredient labels before purchasing a product with PAL (Table 2). Less than two in ten (19%) felt very confident and 60% were somewhat confident in the accuracy of ingredient information on pre-packaged foods and whatever the variables considered, there was no significant differences between the two groups of consumers (Table 2). Efforts were often required by the allergic community to understand labels since 17% of surveyed participants contacted manufacturers always or most of the time, and consumers from the 3rd party panel (28%) were more likely to contact manufacturers than members of the FAC database (12%). Female gender was also associated with a higher likelihood of contacting manufacturers (Table 2). In addition, 77% of consumers were more likely to trust domestic products compared to products manufactured outside of Canada.

***PAL***: Results of the interpretation of PAL by consumers are shown in Table 3. Among respondents, 49% consider that a PAL means a low level of allergen may or may not be in the product but 34% consider the allergen is not or not likely in the product.

Respondents’ knowledge and practices about PAL are presented in Table 4. PAL was considered a very useful tool by a little more than one in two consumers (53%, no significant difference between the two groups of consumers for both criteria) and 45% said that a product without PAL could even be considered unsafe. However, 54% of them had purchased products that have a PAL statement on at least one occasion (significantly higher in panel (80%) than in FAC members (43%); *p* < 0.001) (Table 4). In addition, products with blanket statements (i.e., PAL that includes all or most priority allergens) were considered unsafe by 2/3 of consumers (significant difference between FAC members and Panel members).

Factors impacting a consumer’s decision to buy a product with a PAL statement are detailed in Table 5. The “perception of the likelihood that the allergen is actually present in the product” was the most cited factor (54%), followed by the factor “no prior reaction to the product (product previously consumed without any reaction)” (52%) for all respondents. These two factors were also the main ones taken into account for the FAC group (66% and 65%, respectively) but not for the third-party group (37% vs. 36%). “Type of allergen” (43%) and “Severity of reaction to that allergen” (39%) were the top two factors cited by the third-party panel regarding factors impacting their decision to purchase a product carrying a PAL statement (Table 5).

**Figure 1:**
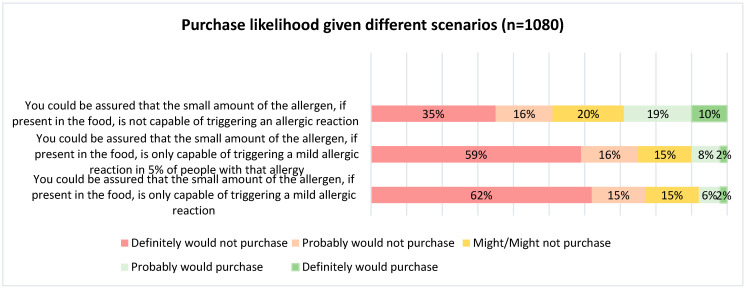
Purchase likelihood of consumers given different scenarios.

**Figure 2:**
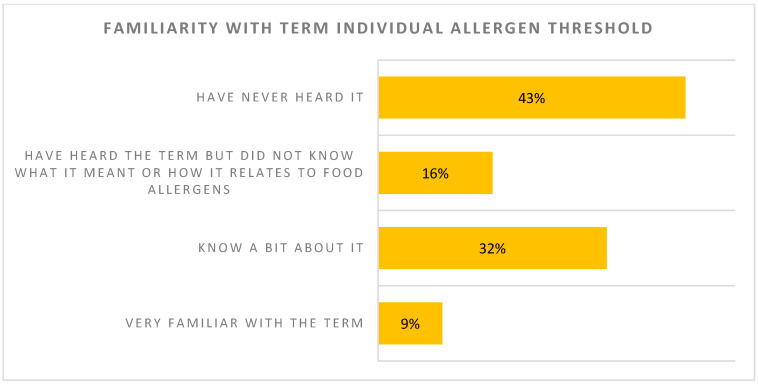
Familiarity with term “Individual allergen threshold”.

**Figure 3:**
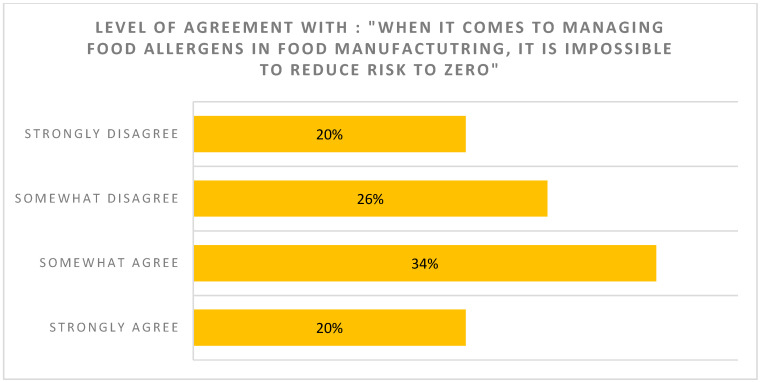
Perspective on ability to reduce risk to zero.

***Allergen thresholds***: Consumers were reluctant to buy foods with even a small amount of their allergen in the product, even if they could be assured that the small amount of allergen is highly unlikely to trigger an allergic reaction in the vast majority (Figure 1). In total, 59% of surveyed individuals had not heard the term “individual allergen threshold” or had heard the term but did not know what it meant (Figure 2). For 54% of respondents, their perception was that it is impossible to reduce the risk to zero when it comes to managing food allergens in food manufacturing (Figure 3). The majority of respondents (83%) did, however, agree that a standardized, scientific, risk-based approach to PAL is needed, and this would make PAL less confusing (70%), more useful (78%) and increase confidence among those managing food allergies (76%) when purchasing food products 

### 3.2. Allergist Survey

Characteristics of surveyed allergists are detailed in Table 6. Sixty-three allergists responded to the survey representing approximately 29% of allergists in Canada (63/219) [24]. The majority of responders were from Ontario (39%) and Quebec (35%) and had more than 20 years of experience (42%). Fifty-one percent cared for both adults and children. In addition, surveyed allergists worked full or part-time in an academic hospital centre (51%), a non-academic community hospital centre (21%), and private practice (68%). Thirty-five percent of allergists worked in multiple clinical settings. Thirty-eight percent worked exclusively in private practice. Most allergists surveyed (82%) performed oral food challenges (OFCs) more than once a week. This was not significantly different when comparing different types of practice (73% in private practice vs. 89% in academic/mixed practices, *p* > 0.05).

Half of responders thought PAL was not useful in its current form. Many allergists supported the consumption of foods with PAL in some of their patients (69% for adults and 83% for children) based on multiple considerations including comorbidities, FA severity, previous tolerance of foods with PAL, and OFC threshold (Table S1 in Supplementary Materials). Recommendations to avoid foods with PAL were generally not influenced by skin prick test size or specific IgE levels (23% yes, 54% no, 23% it depends), but rather by OFC reactivity threshold (66%), although only 19% used a specific cut-off value to avoid foods with PAL (100 mg was cited by three respondents from Quebec). In addition, the type of food allergen listed in the PAL statement did not influence allergists’ recommendations regarding PAL in two-thirds of cases. For those who answered that the type of food did influence their decision (21/56; 37%), foods with PAL to eggs (71%), milk (67%), soy (67%) were more frequently allowed than peanuts (24%), tree nuts (28%), mustard (38%), fish (24%) and crustaceans (38%) (*p* < 0.05).

Fifty-eight percent of allergists agreed that PAL labelling should not be used when the presence of a cross-contact allergen is unlikely to trigger an allergic reaction in the vast majority of patients (i.e., below the ED_05_). However, 23% disagreed and 19% were unsure, with some mentioning that the most sensitive patients are those who will most benefit from highly restrictive PAL. The majority of allergists (75%) were prepared to perform a single-dose OFC to assess the tolerance to a small defined quantity of an allergen for the purpose of determining whether or not a patient could safely consume certain foods with PAL. However, all clinicians anticipated several difficulties in providing guidance/information to their patients with the aforementioned approach (Table S2). The key findings of the allergist survey are summarized in Table 7 and detailed survey responses can be found in the Supplementary Materials.

## 4. Discussion

Allergen cross-contacts can occur at several steps of the food production chain and the amount of allergen potentially present in the final food is variable. This is the reason why Health Canada recommends avoidance by food allergic consumers of all food and products that carry a precautionary statement warning [25]. However, with the notable exception of milk, for which there is both high prevalence and concentration in chocolate-based and other food products [26,27,28], several studies have shown that many food products with PAL contain no or only very low amounts of allergens [29]. Recent Canadian studies reported that 90% of products with a PAL for egg, peanut and hazelnut had absent or very low concentrations of allergens (i.e., below the limit of quantification of detection tests) [26,30]. For hazelnut, the remaining 10% had a relatively high mean concentration (range 0.4 to 2167 ppm) but this is an allergen that presents with a risk of heterogeneous (variable amounts of allergen residues in the same product batch) and high-concentration contamination [30].

As reported in previous studies [31,32], this overuse of PAL and possible past experiences by some of consuming foods with PAL without any adverse reactions probably explain why our study reported that more than half of Canadian allergic consumers purchase products that have a PAL statement. This is a higher reported consumption of products with PAL by the community with FA compared to 2014 when only 15 to 37% declared consuming products with a precautionary statement [33]). This attitude might have been partly reinforced by the medical community (mainly allergists) allowing foods with PAL in some patients who have a high reactivity threshold [34].

As in several other studies [13,33,35], our results show that there is a lack of consensus on how consumers who manage food allergies interpret precautionary statements. Most commonly, they assume that precautionary allergen statements such as “may contain” mean that a low level of allergens may or may not be in the product. One-third (34%) of consumers (no statistical difference between the two groups) believe that manufacturers use PAL for legal protection purposes, which indirectly means that they do not trust PAL and that the product can be consumed in many cases (Table 3). However, even with uncertainties regarding this labelling, more than half of consumers in this survey consider PAL as a very useful tool and there is no significant difference between our two groups of consumers regarding this point; seeing PAL on food products makes them feel more confident that the company is managing allergens. In fact, some consumers dealing with food allergies have concerns about the safety of food products that do not contain any PAL believing they may not be safe without any differences reported between the FAC members and the third-party panel. Physicians are more sceptical since half think PAL is not useful in its current form. Importantly, the combination of PAL overuse by manufacturers with potential heterogeneous presence of allergens and the increased consumption of pre-packaged foods with PAL by consumers, allowed in some cases by clinicians, might lead to allergic incidents after consumption of these foods. Overall, FAC members are better informed about products with a PAL than panel members and adopt more cautious behaviours regarding the purchase of pre-packaged food products with PAL.

Respondents to the third-party panel, for example, were less likely to report reading the label and more likely to have purchased products with PAL. Individuals registered with FAC are more likely than those in the third-party panel to be parents of a child with food allergies or to have multiple allergies (Table 1). This likely explains why they would be generally more involved in managing their allergic condition and are probably more informed about interpreting labelling statements compared to those not registered with allergic consumer associations.

The ad hoc Joint FAO/WHO Expert Consultation proposed reference doses for common allergens in combination with an allergen risk assessment to inform the decision whether or not to apply a PAL statement on pre-packaged food products (FAO/WHO work group (WG), 2021) [15,16,36,37]. However, when we questioned consumers about reference doses (called thresholds in the questionnaire), most were not familiar with the concept, even if they understood that the amount of allergen that triggers an allergic reaction differs between individuals and to a lesser extent with the type of allergen and amount consumed. When it comes to managing food allergens in food manufacturing, allergic consumers are divided as to whether they believe it is possible to reduce the risk to zero, but interestingly, they are generally hesitant to buy foods with even a small amount of the allergen in the product unlikely to trigger an allergic reaction. Even if they could be assured that the allergen content of a food with PAL would only cause a mild allergic reaction in a small percentage of people with the allergy, they were unlikely to buy it. On the other hand, some patients accept taking risks with PAL products since they assume that, generally, the allergen is unlikely to be there. The lack of willingness to consume even a low level of allergen is not surprising considering long-standing health care provider recommendations on strict allergen avoidance as well as education on the unpredictability of severe reactions that can occur even to small amounts. Furthermore, since most consumers are unfamiliar with the concept of threshold, it is understandable that they may not want to purchase foods containing a small amount of the allergen. This is an important observation if stakeholders want to contemplate how to integrate reference threshold doses in the management of PAL and to communicate allergen risk assessment to consumers in a regulatory framework as proposed by the FAO/WHO WG. Educational measures would be required if reference doses were to be used as a future allergy risk management tool. These measures would have to take into account the complexity of communicating probabilistic risk and the psychological factors that contribute to zero-risk expectations [13,38,39,40].

From the allergists’ perspective, reaction threshold on an OFC was perceived as an important deciding factor when guiding patients towards avoidance or introduction of foods with PAL. Interestingly, a 100 mg peanut protein reaction threshold was empirically used by some clinicians to allow introduction of foods with PAL, although most did not specify a specific cut-off. A reference dose of 2 mg of peanut protein was proposed by the FAO/WHO WG to decide whether a PAL is required on a pre-packaged product (FAO/WHO WG, 2021) [15,16,36,37], knowing that this amount of peanut corresponds to a quantity of peanut protein slightly lower than the ED_05_ [18,41]. The implication is that “high threshold” reactors might safely consume these products with PAL in the context where recommendations proposed by the FAO/WHO WG apply, and that a low level of allergens in prepackaged foods with PAL could be assured. If PAL would mean a low level of allergen, a single dose OFC at a defined threshold would be an interesting avenue to allow introduction of foods with PAL in patients who do not react at this dose, thereby increasing available food options. On the other hand, if the food challenge is positive, these patients would be advised to pursue avoidance of foods with PAL. Most allergists from the survey were open to performing single-dose food challenges to stratify patients’ risk regarding PAL introduction, as previously proposed by some clinicians in clinical practice [42,43]. The most significant obstacles foreseen with this approach included limited access to OFCs in Canada and variability of thresholds with external cofactors such as exercise, sleep deprivation or concomitant use of drugs [42].

A limitation of the consumer survey was that clinical confirmation of food allergy by a medical practitioner relied on an online questionnaire completed by the respondent. However, this approach produced less biased results than a simple question about food allergy diagnosis, which would not have ruled out erroneous self-diagnosis. Furthermore, the majority (72%) of respondents were members of Food Allergy Canada, who—as our results showed—were more aware of allergic diseases and better informed about food allergen labelling than non-member consumers. However, the multivariate analysis made possible to take into account significant differences observed for some variables between the two groups of consumers. Regarding the limitations of the allergist survey, there is a possible selection bias with mainly CSACI members participating (i.e., no survey of non-members who are largely community based) although provincial associations were also contacted. Though there is an overrepresentation of Ontario and Quebec, this is where the majority of allergists in Canada are concentrated. Finally, the large representation of full or part-time academic practitioners in the sample could explain the high rate of clinicians performing OFCs in the survey.

As proposed by the FAO/WHO WG in 2022 [16], the use by manufacturers of reference doses combined with allergen risk assessments for the management of PAL could be an option which would allow a possible reduction in the use of PALs on pre-packaged food products and would contribute to reducing the reported variability of allergen concentrations in products with PAL. However, while this approach would standardize and significantly improve the approach manufacturers take to the application of PAL, our study shows it is challenging for the allergic consumer. With limited understanding of the concept of thresholds, including familiarity with their own threshold of reactivity, they are not willing to buy foods that contain even a small amount of allergen in the product, which might only cause mild reactions in a small minority of allergic individuals. Allergic consumers are not comfortable with the concept of reference doses but when they learn the current uncertainties and lack of regulation regarding the use of PAL, they become concerned with the status quo. With almost half of the respondents believing zero risk is possible, there is a lack of understanding of the manufacturing process and the approach some companies take to mitigate risks. This makes it even more difficult to then have consumers contemplate how set reference doses would create a better system to determining PAL. Furthermore, if these international recommendations were to be regulated in Canada, transparency and consumer education on this approach is required to explain the rare possibility of food allergic reactions in the context of an unlabelled unintended presence of allergen in a food product. In addition, for the 5% with a reactivity threshold below the ED_05_, the FAO/WHO recommendations may not improve their QoL because the absence of PAL could mean either an absence of the allergen or an amount of allergen sufficient to trigger an allergic reaction in these very sensitive individuals. For these patients, options will have to be proposed before moving forward with the FAO/WHO recommendations.

Given the complexity of introducing and adopting a risk-based approach to the application of PAL with set reference doses, a collaborative approach between regulators, industry, allergists and patients is necessary to determine the best way to move towards implementation. Allergists and patient organizations can begin by helping patients understand threshold concepts and a risk management approach to PAL. Pilot implementation of such an approach might be another option to support consumer acceptance. However, in an environment where the true risk is unknown and variable, waiting for these concepts to be unilaterally understood has risk considering the continued proliferation in the use of PAL. Of all the possible short-term options, we believe that a risk-based approach for PAL mainly supported by GMPs, combined with guidance for the industry would ultimately help allergic consumers, in a shared decision with their allergists, to better deal with PAL. Support of this approach by regulators as well as further information and education around allergen risk assessment for consumers but also for clinicians could give more confidence in PAL for the majority of consumers with food allergies; this would be a significant improvement compared to what they currently face.

## 5. Conclusions

More and more patients with FA consume foods with PAL in Canada and many allergists authorize consumption of these foods by their patients although these products remain potentially unsafe in the absence of a risk-based approach conducted by most manufacturers. Overall, allergic consumers registered in the FAC database are more inclined to systematically read ingredient labels than allergic consumers from the general population. Furthermore, when purchasing a product with a PAL statement, the reasons behind this decision may be significantly different depending on the consumer groups. While the majority of all consumer’s groups claim to want a more systematic risk-based labelling system, many are reluctant to buy foods with even a small amount of allergen that would be unable to trigger an allergic reaction in the vast majority of them. The risk-based system where a small amount of allergen, safe for the majority (95%) of allergic consumers, could be unintentionally present but not indicated on labels remains currently a challenging approach. Such an approach is not fully supported by the allergic community and problematic for the minority of very sensitive individuals (5%) for whom products without PAL will not be accessible. Thus, a multi-stakeholder approach including additional options for highly sensitive individuals coupled with greater consumer and allergist education on risk assessment concepts is needed when considering allergen thresholds for PAL application. This is of critical importance as this strategy would ultimately provide more clarity towards the application of PAL and benefit the majority of patients.

## Figures and Tables

**Table 1:** Characteristics of respondents (i.e., allergic consumers).

	All Respondents		FAC Database		3rd Party Panel		Two-Sided z-Test Results *p*_adj._	
	N	%	N	%	N	%		
Sample Size	1080	100%	774	72%	306	28%	n/a	
Adults with FA	539	50%	318	41%	221	72%	<0.001	
Parent of a child with FA—Age of child	541	50%	456	59%	85	28%	<0.001	
[0–5]	130	24%	106	23%	24	28%	0.399	
[6–9]	95	18%	75	16%	19	22%	0.262	
[10–12]	79	15%	64	14%	15	18%	0.464	
[13–17]	137	25%	120	26%	16	19%	0.216	
[18+]	99	18%	88	19%	11	13%	0.238	
Single vs. Multiple FA								
Single	334	31%	202	26%	131	43%	<0.001	
Multiple	746	69%	571	74%	175	57%	<0.001	
Priority food allergens (in Canada)								
Peanuts	581	54%	489	63%	91	30%	<0.001	
Tree nuts	545	50%	477	62%	68	22%	<0.001	
Eggs	211	20%	173	22%	38	12%	<0.001	
Milk	211	20%	141	18%	70	23%	0.143	
Shellfish-Crustaceans	180	17%	125	16%	55	18%	0.518	
Shellfish-Molluscs	161	15%	106	14%	54	18%	0.167	
Wheat and Triticale	131	12%	84	11%	47	15%	0.079	
Sesame	131	12%	111	14%	20	7%	0.001	
Fish	119	11%	81	10%	37	12%	0.500	
Soy	93	9%	71	9%	22	7%	0.381	
Mustard	49	5%	35	5%	14	5%	0.970	
Sulphites	68	6%	45	6%	23	8%	0.381	
Non-Priority food allergens (in Canada)								
Fruits	115	11%	87	11%	29	9%	0.466	
Vegetables	68	6%	58	8%	10	3%	0.022	
Who Diagnosed								
Allergist	821	76%	659	85%	162	53%	<0.001	
Family physician	334	31%	169	22%	165	54%	<0.001	
Emergency department physician	207	19%	171	22%	36	12%	<0.001	
Paediatrician	95	9%	70	9%	24	8%	0.569	
Gastroenterologist	56	5%	40	5%	17	6%	0.817	
Time since FA diagnosis								
Less than 6 months ago	37	3%	20	3%	17	6%	0.033	
6 to 11 months ago	80	7%	53	7%	27	9%	0.356	
1 to 2 years ago	131	12%	77	10%	55	18%	<0.001	
3 to 5 years ago	142	13%	94	12%	48	16%	0.195	
6 to 10 years ago	182	17%	123	16%	60	20%	0.216	
More than 10 years ago	508	47%	408	53%	100	33%	<0.001	
Epinephrine auto injector prescription								
Yes	885	82%	706	91%	179	58%	<0.001	
No	194	18%	67	9%	127	42%	<0.001	
Oral food challenge done								
Yes, in the past 3 years	247	23%	157	20%	89	29%	0.005	
Yes, more than 3 years ago	260	24%	166	21%	93	30%	0.005	
No	573	53%	450	58%	123	40%	<0.001	

Multiple responses were accepted. FA: Food allergy data are presented as percentage of individuals who have answered affirmatively to each, broken down by each characteristic. Due to the weighting used, the sums may not correspond exactly to the unweighted starting headcount. In addition, all figures have been rounded after weighting. The [18+] age category corresponds to adults who live with their parents; they were considered ‘adults’ in the following tables and in the rest of the analysis. n/a: non applicable.

**Table 2:** Consumers’ practices about food labelling—total sample N = 1080.

Label	N	%	Explanatory Variable(s)	Categories	N	%	Logistic Regression			
							Univariate		Multivariate	
							OR [95%CI]	*p*_adj._	OR [95%CI]	*p*_adj._
Always reading ingredient labels	627	58%	Sample	3rd Party Panel	98	32%	Ref.			
				FAC Database	526	68%	4.59 [3.45–6.10]	<0.001	2.93 [1.98–4.34]	<0.001
			Age	>54	81	46%	Ref.			
				<18	286	65%	2.17 [1.52–3.10]	<0.001	2.39 [1.44–3.99]	0.004
				[18–34]	132	54%	1.38 [0.93–2.03]	0.140	1.80 [1.06–3.07]	0.066 *
				[35–54]	125	59%	1.70 [1.14–2.55]	0.015	2.91 [1.70–4.97]	<0.001
			Gender	Male	101	47%	Ref.			
				Female	505	61%	1.78 [1.32–2.41]	<0.001	0.95 [0.64–1.42]	0.831
			Income	<USD 100 K	176	46%	Ref.			
				USD 100 K+	271	64%	2.10 [1.58–2.79]	<0.001	1.28 [0.91–1.79]	0.229
			Epinephrine Prescribed	No	66	34%	Ref.			
				Yes	558	63%	3.32 [2.40–4.61]	<0.001	1.69 [1.08–2.64]	0.052 *
			Food Allergy Type	Single	170	51%	Ref.			
				Multiple	455	61%	1.54 [1.19–2.00]	0.002	1.26 [0.89–1.78]	0.253
			Diagnosed by Allergist	No	103	40%	Ref.			
				Yes	526	64%	2.62 [1.97–3.50]	<0.001	1.57 [1.06–2.31]	0.055 *
Very confident of accuracy of ingredient information	203	19%	Sample	3rd Party Panel	86	28%	Ref.			
				FAC Database	116	15%	0.46 [0.33–0.63]	<0.001	0.51 [0.33–0.80]	0.013
			Age	>54	28	16%	Ref.			
				<18	66	15%	0.93 [0.58–1.52]	0.853	0.99 [0.54–1.84]	0.986
				[18–34]	56	23%	1.61 [0.97–2.67]	0.092	1.67 [0.91–3.04]	0.180
				[35–54]	53	25%	1.84 [1.10–3.06]	0.029	1.59 [0.88–2.88]	0.207
			Gender	Male	49	23%	Ref.			
				Female	149	18%	0.76 [0.53–1.10]	0.185	1.09 [0.70–1.70]	0.739
			Income	<USD 100 K	81	21%	Ref.			
				USD 100 K+	85	20%	0.95 [0.67–1.35]	0.853	1.30 [0.88–1.93]	0.253
			Epinephrine Prescribed	No	49	25%	Ref.			
				Yes	150	17%	0.63 [0.44–0.91]	0.023	0.74 [0.46–1.19]	0.274
			Food Allergy Type	Single	63	19%	Ref.			
				Multiple	142	19%	0.99 [0.71–1.38]	0.972	1.30 [0.87–1.95]	0.268
			Diagnosed by Allergist	No	57	22%	Ref.			
				Yes	148	18%	0.74 [0.52–1.04]	0.116	0.81 [0.53–1.25]	0.387
Always/most of the time contacting a manufacturer	181	17%	Sample	3rd Party Panel	86	28%	Ref.			
				FAC Database	93	12%	0.35 [0.25–0.49]	<0.001	0.25 [0.15–0.42]	<0.001
			Age	>54	12	7%	Ref.			
				<18	84	19%	2.84 [1.54–5.24]	0.002	3.04 [1.33–6.95]	0.026
				[18–34]	44	18%	2.73 [1.43–5.22]	0.004	2.38 [1.03–5.48]	0.086 *
				[35–54]	40	19%	2.97 [1.54–5.74]	0.002	3.05 [1.34–6.93]	0.026
			Gender	Male	58	27%	Ref.			
				Female	116	14%	0.43 [0.30–0.62]	<0.001	0.56 [0.35–0.90]	0.043
			Income	<USD 100 K	69	18%	Ref.			
				USD 100 K+	76	18%	1.01 [0.70–1.46]	0.972	1.25 [0.80–1.94]	0.387
			Epinephrine Prescribed	No	29	15%	Ref.			
				Yes	150	17%	1.22 [0.79–1.88]	0.450	1.32 [0.75–2.35]	0.387
			Food Allergy Type	Single	50	15%	Ref.			
				Multiple	127	17%	1.15 [0.81–1.63]	0.517	1.44 [0.91–2.28]	0.207
			Diagnosed by Allergist	No	41	16%	Ref.			
				Yes	140	17%	1.05 [0.72–1.53]	0.853	1.45 [0.87–2.43]	0.229

Data are presented as percentage of individuals who have answered affirmatively to each question (compared to those who answered negatively), broken down by each characteristic. Due to the weighting used, the sums may not correspond exactly to the unweighted starting headcount. In addition, all figures have been rounded after weighting. * In some cases, the adjusted *p*-value may appear as non-significant even though the odds ratio (OR) is greater than 1 and the confidence interval (CI) does not include 1. This can occur due to the Benjamini–Hochberg adjustment for multiple comparisons, which controls the false discovery rate. This adjustment makes the test for statistical significance more stringent, particularly when multiple variables are analysed simultaneously. As a result, an observed association that appears statistically significant before adjustment may not meet the stricter threshold for significance once the adjustment is applied.

**Table 3:** Interpretations of PAL by consumers.

	All Respondents		FAC Database		3rd Party Panel		Two-Sided z-Test Results *p*_adj._
	N	%	N	%	N	%	
Sample Size	1080	100%	774	72	306	28	n/a
A low level of allergen is in the product	124	11	59	8	65	21	<0.001
A low level of allergen may or may not be in the product	530	49	404	52	126	41	0.003
The allergen is not likely in the product and precautionary allergen labelling is used by the manufacturer for legal protection	311	29	229	30	82	27	0.452
The allergen is not in the product and precautionary allergen labelling is used by the manufacturer for legal protection	55	5	36	5	19	6	0.452
Don’t know/not sure	59	5	44	6	15	5	0.610

Data are presented as percentage of individuals who have answered affirmatively to each question (compared to those who answered negatively), broken down by each characteristic. Due to the weighting used, the sums may not correspond exactly to the unweighted starting headcount. In addition, all figures have been rounded after weighting. n/a: non applicable.

**Table 4:** Consumers’ knowledge and practices about PAL—total sample N = 1080.

Label	N	%	Explanatory Variable(s)	Categories	N	%	Logistic Regression			
							Univariate		Multivariate	
							OR [95%CI]	*p*_adj._	OR [95%CI]	*p*_adj._
Aware that PAL is voluntary	474	44%	Sample	3rd Party Panel	119	39%	Ref.			
				FAC Database	356	46%	1.32 [1.01–1.73]	0.083	1.20 [0.82–1.76]	0.458
			Age	>54	53	30%	Ref.			
				<18	225	51%	2.35 [1.62–3.42]	<0.001	2.52 [1.52–4.17]	0.003
				[18–34]	103	42%	1.70 [1.13–2.56]	0.025	1.79 [1.06–3.03]	0.089 *
				[35–54]	91	43%	1.71 [1.12–2.60]	0.028	2.62 [1.55–4.41]	0.003
			Gender	Male	103	48%	Ref.			
				Female	356	43%	0.83 [0.61–1.12]	0.306	0.71 [0.49–1.04]	0.163
			Income	<USD 100 K	157	41%	Ref.			
				USD 100 K+	199	47%	1.28 [0.97–1.70]	0.133	1.01 [0.74–1.39]	0.950
			Epinephrine Prescribed	No	62	32%	Ref.			
				Yes	416	47%	1.87 [1.35–2.60]	<0.001	1.49 [0.97–2.30]	0.157
			Food Allergy Type	Single	140	42%	Ref.			
				Multiple	336	45%	1.10 [0.84–1.42]	0.565	1.06 [0.77–1.47]	0.818
			Diagnosed by Allergist	No	98	38%	Ref.			
				Yes	378	46%	1.41 [1.06–1.87]	0.041	1.04 [0.71–1.51]	0.904
PAL considered as very useful	573	53%	Sample	3rd Party Panel	153	50%	Ref.			
				FAC Database	418	54%	1.17 [0.89–1.52]	0.347	0.98 [0.67–1.43]	0.950
			Age	>54	74	42%	Ref.			
				<18	260	59%	2.03 [1.43–2.90]	<0.001	1.95 [1.21–3.12]	0.029
				[18–34]	135	55%	1.70 [1.15–2.52]	0.018	1.46 [0.90–2.37]	0.226
				[35–54]	100	47%	1.23 [0.82–1.84]	0.385	1.06 [0.66–1.72]	0.894
			Gender	Male	107	50%	Ref.			
				Female	447	54%	1.18 [0.87–1.59]	0.369	1.13 [0.78–1.63]	0.644
			Income	<USD 100 K	196	51%	Ref.			
				USD 100 K+	233	55%	1.16 [0.88–1.54]	0.369	0.95 [0.70–1.30]	0.865
			Epinephrine Prescribed	No	89	46%	Ref.			
				Yes	487	55%	1.41 [1.03–1.93]	0.061	1.31 [0.87–1.99]	0.275
			Food Allergy Type	Single	180	54%	Ref.			
				Multiple	388	52%	0.92 [0.71–1.19]	0.608	1.03 [0.75–1.42]	0.904
			Diagnosed by Allergist	No	129	50%	Ref.			
				Yes	444	54%	1.16 [0.88–1.54]	0.369	0.85 [0.59–1.22]	0.475
Products without PAL considered as unsafe	482	45%	Sample	3rd Party Panel	122	40%	Ref.			
				FAC Database	356	46%	1.29 [0.99–1.69]	0.105	1.58 [1.07–2.32]	0.084 *
			Age	>54	76	43%	Ref.			
				<18	211	48%	1.26 [0.89–1.80]	0.280	1.74 [1.07–2.81]	0.089 *
				[18–34]	98	40%	0.89 [0.60–1.31]	0.618	1.40 [0.85–2.31]	0.274
				[35–54]	95	45%	1.09 [0.73–1.64]	0.714	1.71 [1.04–2.81]	0.092 *
			Gender	Male	75	35%	Ref.			
				Female	397	48%	1.71 [1.25–2.34]	0.002	1.44 [0.99–2.09]	0.141
			Income	<USD 100 K	184	48%	Ref.			
				USD 100 K+	174	41%	0.75 [0.56–0.99]	0.083	0.62 [0.45–0.86]	0.024
			Epinephrine Prescribed	No	87	45%	Ref.			
				Yes	390	44%	0.96 [0.70–1.31]	0.847	0.72 [0.47–1.10]	0.226
			Food Allergy Type	Single	160	48%	Ref.			
				Multiple	321	43%	0.83 [0.64–1.08]	0.240	0.78 [0.56–1.08]	0.226
			Diagnosed by Allergist	No	114	44%	Ref.			
				Yes	370	45%	1.04 [0.78–1.37]	0.847	1.11 [0.76–1.60]	0.722
Products with blanket statement considered as unsafe	719	67%	Sample	3rd Party Panel	141	46%	Ref.			
				FAC Database	580	75%	3.74 [2.82–4.95]	<0.001	2.97 [2.00–4.41]	<0.001
			Age	>54	109	62%	Ref.			
				<18	330	75%	1.81 [1.24–2.65]	0.006	2.01 [1.20–3.39]	0.039
				[18–34]	159	65%	1.12 [0.74–1.68]	0.658	1.15 [0.68–1.93]	0.722
				[35–54]	116	55%	0.69 [0.46–1.05]	0.133	0.98 [0.59–1.64]	0.950
			Gender	Male	114	53%	Ref.			
				Female	579	70%	2.09 [1.53–2.85]	<0.001	1.38 [0.93–2.03]	0.215
			Income	<USD 100 K	238	62%	Ref.			
				USD 100 K+	284	67%	1.28 [0.95–1.72]	0.159	0.77 [0.54–1.10]	0.246
			Epinephrine Prescribed	No	95	49%	Ref.			
				Yes	620	70%	2.49 [1.81–3.43]	<0.001	1.41 [0.91–2.19]	0.226
			Food Allergy Type	Single	207	62%	Ref.			
				Multiple	515	69%	1.32 [1.01–1.74]	0.083	1.24 [0.87–1.76]	0.321
			Diagnosed by Allergist	No	155	60%	Ref.			
				Yes	567	69%	1.49 [1.11–1.99]	0.018	0.76 [0.51–1.13]	0.263
Buying products with PAL at least some of the time	580	54%	Sample	3rd Party Panel	245	80%	Ref.			
				FAC Database	333	43%	0.19 [0.14–0.27]	<0.001	0.28 [0.18–0.42]	<0.001
			Age	>54	95	54%	Ref.			
				<18	203	46%	0.72 [0.51–1.02]	0.110	1.40 [0.85–2.32]	0.274
				[18–34]	127	52%	0.91 [0.62–1.35]	0.696	1.46 [0.86–2.48]	0.257
				[35–54]	152	72%	2.21 [1.45–3.37]	<0.001	2.20 [1.28–3.78]	0.024
			Gender	Male	146	68%	Ref.			
				Female	414	50%	0.46 [0.34–0.64]	<0.001	0.63 [0.42–0.95]	0.089
			Income	<USD 100 K	253	66%	Ref.			
				USD 100 K+	208	49%	0.50 [0.37–0.66]	<0.001	0.69 [0.49–0.96]	0.089
			Epinephrine Prescribed	No	154	79%	Ref.			
				Yes	425	48%	0.25 [0.17–0.36]	<0.001	0.43 [0.26–0.70]	0.005
			Food Allergy Type	Single	184	55%	Ref.			
				Multiple	395	53%	0.90 [0.70–1.17]	0.527	1.38 [0.98–1.95]	0.157
			Diagnosed by Allergist	No	160	62%	Ref.			
				Yes	419	51%	0.65 [0.49–0.86]	0.007	1.58 [1.04–2.39]	0.089 *

Data are presented as percentage of individuals who have answered affirmatively to each question (compared to those who answered negatively), broken down by each characteristic. Due to the weighting used, the sums may not correspond exactly to the unweighted starting headcount. In addition, all figures have been rounded after weighting. * In some cases, the adjusted *p*-value may appear as non-significant even though the odds ratio (OR) is greater than 1 and the confidence interval (CI) does not include 1. This can occur due to the Benjamini-Hochberg adjustment for multiple comparisons, which controls the false discovery rate. This adjustment makes the test for statistical significance more stringent, particularly when multiple variables are analysed simultaneously. As a result, an observed association that appears statistically significant before adjustment may not meet the stricter threshold for significance once the adjustment is applied.

**Table 5:** Factors impacting consumer’s decision to buy a product with a PAL statement.

	All Respondents		FAC Database		3rd Party Panel		Two-Sided z-Test Results *p*_adj._
	N	%	N	%	N	%	
Sample Size	1080	100%	774	72	306	28	n/a
Your perception of the likelihood of the allergen actually being in the product	312	54	222	66	90	37	<0.001
No prior reactions to product (consumed product before without any reactions)	304	52	217	65	87	36	<0.001
Type of allergen(s)	279	48	173	51	106	43	0.071
Information provided directly from manufacturer	249	43	174	52	75	31	<0.001
Severity of reaction to that allergen(s)	244	42	148	44	96	39	0.286
Whether another similar product is available without a precautionary allergen label	205	35	137	41	68	28	0.002
Advice from allergist, doctor, or other health professional	191	33	108	32	83	34	0.636
Eating location (e.g., where the product will be eaten such as at home vs. outside the home)	175	30	117	35	58	24	0.006
The cost (e.g., the product with the precautionary allergen label is lower priced)	72	12	25	7	47	19	<0.001

Data is presented as percentage of individuals who have answered affirmatively to each question (compared to those who answered negatively), broken down by each characteristic. Due to the weighting used, the sums may not correspond exactly to the unweighted starting headcount. In addition, all figures have been rounded after weighting. n/a: non applicable.

**Table 6:** Allergists’ survey responder characteristics [expressed in % and (n)]—total sample N = 57.

Province of Practice	
Alberta, Manitoba, Saskatchewan	8.77% (n = 5)
British Columbia	8.77% (n = 5)
Nova Scotia	8.77% (n = 5)
Ontario	38.6% (n = 22)
Quebec	35.1% (n = 20)
Years of experience	
<1 year to 5 years	28.07% (n = 16)
6–10 years	19.3% (n = 11)
11–20 years	10.53% (n = 6)
20 years or more	42.1% (n = 24)
Type of patients seen	
Adults	14.04% (n = 8)
Children	35.09% (n = 20)
Both adults and children	50.88% (n = 29)
Type of practice *	
Academic	50.88% (n = 29)
Community Hospital Centre	21.05% (n = 12)
Private Practice	68.42% (n = 39)
Mixed Private practice and Academic	35% (n = 27)
OFCs performed	
Daily	33.3% (n = 19)
Weekly to Once a month	52.6% (n = 30)
Rarely (i.e., a few times a year) or Never	14.04% (n = 8)

* multiple responses accepted as 35% (20/57) of allergists practicing in >1 clinical setting. Only 57 out of 63 allergists completed this part of the survey.

**Table 7:** Key messages and differences between consumer and allergist surveys.

Key Messages of Consumer Survey	Key Messages of Allergist Survey
More than half of consumers (53%) consider that PAL is a useful tool. However, they find it confusing in its current form. Seeing PAL statements on food products makes them feel that the manufacturer is more aware of allergens and is taking it seriously.	Half of surveyed allergists think PAL is not useful in its current form.
More than half of consumers (54%) will purchase products that have a PAL statement at least on occasion.	The majority of Canadian allergists surveyed allowed foods with PAL in a proportion of patients with IgE-mediated food allergies based on patient-specific factors and shared decision making (69% for adults and 83% for children).
The majority of surveyed individuals (59%) have not heard the term “individual allergen threshold” or have heard the term but did not know what it meant.	Reaction threshold on an oral food challenge is perceived as an important deciding factor when giving recommendations towards avoidance or introduction of foods with PAL.
Consumers are reluctant to buy foods with even a small amount of their allergen in the product, even if they could be assured that the small amount of allergen is not capable of triggering an allergic reaction	Most allergists think PAL should not be too restrictive (PAL should not be used if unlikely to trigger reaction in the majority of patients)
	Surveyed allergists are open to perform single-dose oral food challenges to stratify low-risk patients who could benefit from PAL introduction, although obstacles are mentioned.

## Data Availability

All data generated or analysed during this study are included in this published article and its Supplementary Materials.

## Supplementary Materials

The following supporting information can be downloaded at: https://www.mdpi.com/article/10.3390/nu17091556/s1, File S1: Food Allergy Canada PAL Research Questionnaire. File S2: Allergist Survey Questions. Table S1: Factors taken into account when allowing patients to introduce foods with precautionary allergen labelling (PAL). Table S2: Challenges foreseen by allergists in providing guidance on precautionary allergen labelled products based on a single-dose oral food challenge.
