# Supplementary material for: A Survey of Allergic Consumers and Allergists on Precautionary Allergen Labelling: Where Do We Go from Here?
Source: Nutrients. 2025 Apr 30;17(9):1556. doi: 10.3390/nu17091556 (PMC12073677; doi:10.3390/nu17091556)
Supplement: Supplementary file 1 [file nutrients-17-01556-s001.zip › Supplementary File S1.pdf]

FOOD ALLERGY CANADA PAL RESEARCH

Questionnaire (FAC-1478)

FINAL November 19, 2021

Project Specs

Methodology: Online survey using FAC's database & a third-party panel.

An email was sent out to all FAC members inviting them to participate in the survey. No compensation / incentive was provided to Food Allergy Canada respondents.

The remaining respondents were sourced through 3 third-party online research panels: Dynata, Disqo and Logit. These panels have hundreds of thousands of panel members and are used exclusively for research purposes. Invitations were sent out randomly to Canadian panel members 18+ in batches until we achieved our sample size. Incentives for panel members are electronic / digital, whereby those who complete the survey in full receive points from the panel provider. These points can then be redeemed for gift cards (similar to a loyalty program).

Geographic scope: National, including Quebec.

Sample size: 1000 completes, broken out as follows

- 750 completes are sourced from FAC's database
- 250 completes through a third party panel, broken out as follows (regional distribution for panel sample is based on population distribution)
    - 30 completes in BC
    - 40 completes in Prairies
    - 100 completes in Ontario
    - 60 completes in Quebec
    - 20 completes in Atlantic Canada

Qualifiers:

- Q1 (panel respondents) – Must reside in BC, Prairies, Ontario, Quebec, or Atlantic Canada – Watch quotas.
- Q2 – (panel respondents) - Must be 18+
- Q3 – Must have sole or shared responsibility for grocery shopping
- Q5 – Must have a food allergy or have a child with a food allergy
- Q8 – Food allergy was diagnosed / confirmed by a medical professional (pediatrician, family physician (GP), allergist, emergency room doctor, etc.)

Questionnaire

SECTION 1: Screener & Profiling

1. In which province or territory do you currently reside?

British Columbia	❒
Alberta	❒
Manitoba	❒
Saskatchewan	❒
Ontario	❒
Quebec	❒
Newfoundland & Labrador	❒
Nova Scotia	❒
New Brunswick	❒
Prince Edward Island	❒
Nunavut	❒
Yukon	❒
Northwest Territories	❒
None of the above	❒

[PANEL RESPONDENTS MUST RESIDE IN BC, PRAIRIES, ONTARIO, QUEBEC OF ATLANTIC CANADA; OTHERWISE THANK & TERMINATE]
[WATCH QUOTAS FOR PANEL RESPONDENT]

[TERMINATION MESSAGE FOR PANEL RESPONDENTS TO READ: Thank you for your interest. Unfortunately, this survey is only applicable to those living in BC, the Prairies, Ontario, Quebec of Atlantic Canada]

2. To ensure we reach a wide cross-section of people, into which of the following age ranges do you fall?

Under 18	❒	**[THANK & TERMINATE IF PANEL RESPONDENT]**
18 to 24	❒	
25 to 34	❒	
35 to 44	❒	
45 to 54	❒	
55 to 64	❒	
65 or older	❒	
**[SHOW TO FAC DATABASE ONLY]** Prefer not to answer	❒	

[TERMINATION MESSAGE TO READ: Thank you for your interest. Unfortunately, this survey is only open to adults 18 years of age or older.]

3. When it comes to grocery shopping for your household, would you say…

You are the primary grocery shopper for your household	❑	
You and another household member share the responsibility	❑	
Another member of your household is the primary grocery shopper	❑	**[THANK & TERMINATE]**

[TERMINATION MESSAGE TO READ: Thank you for your interest. Unfortunately, this survey only pertains to individuals who have sole or shared responsibility for their household's grocery shopping.

4. When grocery shopping, how often do you read food ingredient labels?

Always	❑
Most of the time	❑
Occasionally	❑
Rarely	❑
Never	❑

5. Which of the following best describes you?

[ALLOW RESPONDENTS TO CHECK ONE OR BOTH OF THE FIRST 2 OPTIONS OR THE THIRD OPTION]

I have a food allergy	❑
I am a parent of a child with a food allergy	❑
I do not have any food allergies nor do any of my children	❑ **[THANK & TERMINATE]**

[TERMINATION MESSAGE TO READ: Thank you for your interest in this survey. Unfortunately, the questions are only relevant to individuals with food allergies.]

6. RESPONDENT CLASSIFICATION – DO NOT ASK

IF 'I have a food allergy' AT Q5 CLASSIFY AS AN <u>ADULT (with a food allergy)</u>

IF 'I am a parent of a child with a food allergy' AT Q5 <u>CLASSIFY AS A PARENT (of a child with a food allergy)</u>

IF RESPONDENT CHECKS BOTH 'I have a food allergy' AND 'I am a parent of a child with a food allergy' <u>CLASSIFY AS AN ADULT (with a food allergy).</u>

[IF CLASSIFIED AS AN 'Adult' SHOW] We would like you to answer the remainder of this survey based on your experiences and needs as an **adult** with a food allergy.

[IF CLASSIFIED AS A 'PARENT' SHOW] We would like you to answer the remainder of this survey based on your experiences and needs as a **parent of a child** with a food allergy. If you have more than one child with a food allergy, please complete the survey based on the child whose birthday is coming up next. In the case of twins, answer for the older twin.

7. **[IF 'Parent' AT Q6]** Who diagnosed your child's <u>food</u> allergy? If you have more than one child with a food allergy, please answer based on the child whose birthday is coming up next. In the case of twins, answer for the older twin.

[IF 'Adult' AT Q6] Who diagnosed your <u>food</u> allergy?

(Select ALL that apply)

I made the diagnosis myself	❑	
Allergist / Immunologist	❑	**[MUST SELECT AT LEAST ONE OF THESE 4 OPTIONS; OTHERWISE THANK & TERMINATE]**
Family Physician	❑	
Emergency Department Physician	❑	
Pediatrician		
Gastroenterologist	❑	
Other (please specify)________________	❑	

[TERMINATION MESSAGE TO READ: Thank you for your interest. Unfortunately, this survey is only applicable to those who have had a formal food allergy diagnosis.

8. **[IF 'Parent' AT Q6 ASK]** What is the age of your child with a food allergy? Again, if you have more than one child with a food allergy, please answer based on the child whose birthday comes next. In the case of twins, answer for the older twin.

5 years of age or younger	❑
6 to 9	❑
10 to 12	❑
13 to 17	❑
18 years of age or older	❑

9. **[IF 'Parent' AT Q6 ASK]** Which **<u>food(s)</u>** is your child allergic to?

[IF 'Adult' AT Q6 ASK] Which **<u>food(s)</u>** are you allergic to?

(Select ALL that apply)

[SHOW IN ALPHABETICAL ORDER]

- Egg ❑
- Fish (e.g., trout, salmon) ❑
- Milk ❑
- Mustard ❑
- Peanut ❑
- Sesame ❑
- Shellfish - Molluscs (such as oysters, mussels, squid) ❑
- Shellfish - Crustaceans (such as lobster, shrimp, crab) ❑
- Soy ❑
- Sulphites (an additive, not a food) ❑
- Tree nuts (e.g., almonds, Brazil nuts, cashews, hazelnuts, macadamia nuts, pecans, pine nuts, pistachios, walnuts) ❑
- Wheat & triticale ❑
- **[ALWAYS SHOW LAST]** Other foods (please specify)____________________ ❑

10. **[IF 'Parent' AT Q6 & 'Tree nuts' AT Q9 ASK]** Which type of <u>tree nuts</u> is your child allergic to?

[IF 'Adult' AT Q6 & 'Tree nuts' AT Q9 ASK] Which type of <u>tree nuts</u> are you allergic to?

(Select ALL that apply)

- Almonds ❑
- Brazil nuts ❑
- Cashews ❑
- Hazelnuts ❑
- Macadamia nuts ❑
- Pecans ❑
- Pine nuts ❑
- Pistachios ❑

Walnuts ❑
Other (please specify)____________ ❑

11. **[IF 'Parent' AT Q6 ASK]** When was your child first diagnosed with their food allergy? If they have more than one food allergy, please answer based on the allergy that was first diagnosed.

[IF 'Adult' AT Q6 ASK] When were you first diagnosed with your food allergy? If you have more than one food allergy, please answer based on the allergy that was first diagnosed.

Less than 6 months ago ❑
6 to 11 months ago ❑
1 to 2 years ago ❑
3 to 5 years ago ❑
6 to 10 years ago ❑
More than 10 years ago ❑

12. **[IF 'PARENT' AT Q6 ASK]** Has your child been prescribed an epinephrine auto-injector (e.g., EpiPen®, ALLERJECT®, Emerade™)?

[IF 'Adult' AT Q6 ASK] Have you been prescribed an epinephrine auto-injector (e.g., EpiPen®, ALLERJECT®, Emerade™)?

Yes ❑
No ❑

<u>SECTION 2: Current State</u>

Now we'd like to focus on food labelling and ingredient information including **precautionary allergen labelling**, like "may contain" statements on pre-packaged foods**. By pre-packaged foods we mean food products that are packaged by the manufacturer and then sold by a retailer (**e.g., a box of cookies sold in a grocery store).

13. Overall, how confident are you in the accuracy of ingredient information on pre-packaged food?

Very confident ❑
Somewhat confident ❑
Not very confident ❑
Not at all confident ❑
Haven't really thought about it ❑

14. How often, if ever, do you contact a food manufacturer to ask about or clarify the ingredient information provided on a food product?

Always	❑
Most of the time	❑
Occasionally	❑
Rarely	❑
Never	❑

15. Does your level of trust in food labelling and ingredient information differ at all based on size of manufacturer or country of origin?

	Would have more trust in food labelling and ingredient information from a **<u>smaller</u>** food manufacturer	Size of manufacturer makes no difference to me	Would have more trust in food labelling and ingredient information from a **<u>larger</u>** food manufacturer
Size of Manufacturer	❑	❑	❑

	Would have more trust in food labelling and ingredient information from **<u>domestic</u>** products	Country of origin makes no difference to me	Would have more trust in food labelling and ingredient information from **<u>imported</u>** products
Country product is manufactured in	❑	❑	❑

16. As you may know, some food products use **precautionary allergen labelling** such as **"may contain…", "product was manufactured in a facility that also processes…"** or **"product is manufactured on shared equipment with products containing** 'your allergen' **…"** statements.

[IF 'Parent' AT Q6 ASK] If a product does <u>not</u> use any precautionary allergen labelling how safe do you believe it is for your child to eat?

[IF 'Adult' AT Q6 ASK] If a product does <u>not</u> use any precautionary allergen labelling how safe do you believe it is for you to eat?

Very safe	❑
Somewhat safe	❑
Not very safe	❑
Not safe at all	❑

17. When you see precautionary allergen labelling such "may contain" on food packaging, what do you think or assume this means? Please be as specific and detailed as possible.

18. More specifically, which of the following best describes how you interpret a precautionary allergen statement such as "may contain"?

A low level of allergen is in the product	❑
A low level of allergen may or may not be in the product	❑
The allergen is not <u>likely</u> in the product and precautionary allergen labelling is used by the manufacturer for legal protection	❑
The allergen is <u>not</u> in the product and precautionary allergen labelling is used by the manufacturer for legal protection	❑
Don't know / not sure	❑

[SHOW Q19a & Q19b ON SAME SCREEN]

19a. When shopping for food products, how useful do you find precautionary allergen labelling such as "may contain" or "manufactured in a facility that also processes 'your allergen'…"statements?

Very useful	❑
Somewhat useful	❑

Not very useful ❑

Not at all useful ❑

19b. Please explain your response above in as much detail as possible.

20a. Thinking about how often you come across food products with "may contain" statements for allergens you / your household are impacted by , do you believe you have adequate food choice?

Yes ❑

Usually ❑

No ❑

20b. **[IF 'Usually' OR 'No' AT Q20a ASK] For** which food category / food product is it most difficult to find at least one safe option?

21. **[IF ADULT & SELECT 'Fish', 'Tree nuts', Molluscs or 'Crustaceans' AT Q9 ASK. IF RESPONDENT CHECKS MORE THAN ONE OF 'Fish', 'Tree nuts', Molluscs' OR 'Crustaceans' AT Q10 RANDOMLY CHOOSE ONE TO INSERT**] You mentioned you are allergic to **[INSERT ALLERGEN].** How problematic is it for you when a "may contain" label does not break out the specific species or allergen that may be present in the product and instead uses the group name for your allergen category? For example, stating "may contain tree nuts" vs. stating "may contain almonds" or "may contain shellfish", instead of "may contain shrimp"

[IF PARENT & SELECT 'Fish', 'Tree nuts', Molluscs or 'Crustaceans' AT Q9 ASK. IF RESPONDENT CHECKS MORE THAN ONE OF 'Fish', 'Tree nuts', Molluscs' OR 'Crustaceans' AT Q10 RANDOMLY CHOOSE ONE TO INSERT] You mentioned your child is allergic to **[INSERT ALLERGEN].** How problematic is it when a "may contain" label does not break out the specific species or allergen that may be present in the product and instead uses the group name for your child's allergen category? For example, stating "may contain tree nuts" vs. stating "may contain almonds" or "may contain shellfish", instead of "may contain shrimp".

Very problematic ❑

Somewhat problematic ❑

Not very problematic ❑

Not at all problematic ❑

22. **[IF ‘Parent’ AT Q6 ASK]** When buying food products that will be consumed by your child with a food allergy, how often, if ever, do you purchase the following types of products?

[IF ‘Adult’ AT Q6 ASK] When buying food products you will consume, how often, if ever, do you purchase the following types of products?

**[RANDOMIZE]**	Never	Sometimes / Depends on situation	Always
**[ADULT]** Products with a: May contain ‘your allergen(s)’ claim **[PARENT]** Products with a: may contain ‘your child’s allergen(s)’ claim	❑	❑	❑
**[ADULT]** Products with a Manufactured in a facility that processes ‘your allergen(s)’ claim **[PARENT]** Products with a: manufactured in a facility that processes ‘your child’s allergen(s)’ claim	❑	❑	❑
**[ADULT]** Products with a: Manufactured on shared equipment with products containing ‘your allergen(s)’ claim **[PARENT]** Products with a: manufactured on shared equipment with products containing ‘your child’s allergen(s)’ claim	❑	❑	❑
**[ADULT]** Products that have your allergen(s) listed in the ingredients **[PARENT]** Products that has your child’s allergen(s)listed in the ingredients	❑	❑	❑
**[ADULT]** Products containing “free from” statement or illustrations (e.g., a symbol showing the allergen and a line through it) to indicate the allergen you are allergic to is not present **[CHILD]** Products containing “free from” statement or illustrations (e.g., a symbol showing the allergen and a line through it) to indicate the allergen your child is allergic to is not present	❑	❑	❑
Products that have a blanket precautionary labelling statement that includes all or most priority allergens, such as May contain milk, egg, peanut, tree nuts, fish, mustard , wheat etc..	❑	❑	❑
**[IF ‘Milk’ or “Egg’ AT Q10 ASK]** Products containing a vegan claim	❑	❑	❑

23a. **[IF ADULT AT Q6 & CHECK 'Always' or 'Sometimes / Depends on situation' FOR AT LEAST ONE OF THE FIRST <u>3</u> STATEMENTs AT Q22 ASK]** You mentioned in the previous question that you will buy food products that use precautionary allergen labels (e.g., "may contain" or "was manufactured in a facility that also processes…" statements) for an allergen you are allergic to at least occasionally. Please share your approach / decision making around this? For which situations or circumstances will you do this?

[IF PARENT AT Q6 & CHECKS AT 'Always' or 'Sometimes / Depends on situation' FOR AT LEAST ONE OF THE FIRST <u>3</u> STATEMENTS AT Q22 ASK] You mentioned in the previous question that you will buy food products that use precautionary allergen labels (e.g., "may contain" or "was manufactured in a facility that also processes…" statements) for an allergen your child is allergic to at least occasionally.

Please share your approach / decision making around this? For which situations or circumstances will you do this?

23b. **[IF ADULT AT Q6 & CHECKS AT 'Sometimes / Depends on situation' FOR AT LEAST ONE STATEMENT OF FIRST <u>3</u> STATEMENTS Q22 ASK]** What impacts your decision to purchase food products that use precautionary allergen labels (e.g., "may contain" or "was manufactured in a facility that also processes…" statements) for an allergen you are allergic to?

[IF PARENT AT Q6 & CHECKS AT 'Always' or 'Depends on situation' FOR AT LEAST ONE OF THE FIRST <u>3</u> STATEMENTS AT Q22 ASK] What impacts your decision to purchase food products that use precautionary allergen labels (e.g., "may contain" or "was manufactured in a facility that also processes…" statements) for an allergen your child is allergic to?

(Select ALL that apply)

[RANDOMIZE]

No prior reactions to product (consumed product before without any reactions)	❑
Type of allergen(s)	❑
Eating location (e.g., where the product will be eaten such as at home vs outside the home)	❑
Advice from allergist, doctor, or other health professional	❑
Information provided directly from manufacturer	❑
Your perception of the likelihood of the allergen actually being in the product (e.g., "May contain peanuts" on a grape popsicle product)	❑
Severity of reaction to that allergen(s)	❑
Whether another similar product is available without a precautionary allergen label	❑

The cost (e.g., the product with the precautionary allergen label is lower priced) ❑

[ALWAYS SHOW LAST] Other (please specify)___________ ❑

24a. When shopping for food, how often do you come across products with blanket precautionary labelling statements that includes all or most priority allergens, such as "May contain milk, egg, peanut, tree nuts, fish, mustard, wheat etc.. "

All the time ❑

Often ❑

Occasionally ❑

Rarely ❑

Never ❑

24b. **[DO NOT ASK IF 'Never' AT Q24a]** When you come across blanket precautionary labelling statements such as "may contain milk, egg, peanut, tree nuts, fish, mustard, wheat etc" what do you assume that it means?

24c. **[IF 'Parent' AT Q7 AND NOT 'Never' AT Q24a]** If a product uses a blanket precautionary label such as "may contain milk, egg, peanut, tree nuts, fish, mustard, wheat etc" how safe do you believe it is for your child to eat?

[IF 'Adult' AT Q7 & NOT 'Never' AT Q24a ASK] If a product uses a blanket precautionary label such as "may contain milk, egg, peanut, tree nuts, fish, mustard, wheat etc" how safe do you believe it is for you to eat?

Very safe ❑

Somewhat safe ❑

Not very safe ❑

Not safe at all ❑

Unsure ❑

25. When it comes to precautionary allergen labelling such as “may contain” or “manufactured in a facility that also processes ‘your allergen’...” statements, which of the following, if any, do you believe or assume to be true?

(Select ALL that apply)

**[RANDOMIZE]**	
Precautionary allergen labelling such as “may contain” labelling by food manufacturers is required by law.	❑
Precautionary allergen labelling is regulated by Health Canada.	❑
Precautionary allergen labelling language differs depending on the quantity of allergen that is present in the product (e.g., "May contain traces” has a smaller amount of allergen present than a "May contain” statement).	❑
Scientific data on how much of an allergen would cause an allergic reaction is being used by manufacturers to determine the need for precautionary allergen labelling	❑
Manufacturers determine the need for precautionary allergen labelling based on their own internal allergen management practices and risk assessments.	❑
Precautionary allergen labelling is voluntary, and manufacturers are not required to use it.	❑
Precautionary allergen labelling is used by food manufacturers for legal protection only.	❑
Precautionary allergen labelling is used when food manufacturers are unable to avoid cross-contamination with allergens in their manufacturing processes.	❑
**[ALWAYS SHOW LAST]** None of the above	❑

26. To the best of your knowledge, does the amount of allergen that triggers an allergic reaction differ by…?

**[RANDOMIZE]**	Yes	No	Don't know
Individual	❑	❑	❑
Type of allergen	❑	❑	❑
Amount consumed	❑	❑	❑

27. "Individual Allergen threshold" is a term used by some to mean the lowest amount of an allergen that could trigger an allergic reaction in a specific individual, and below that level, no reaction is expected. How familiar are you with this term?

Have never heard it ❑

Have heard the term but didn't know what it meant or how it relates to food allergens ❑

Know a bit about it ❑

Very familiar with the term ❑

28. To what extent do you agree or disagree with the following statement:

When it comes to managing food allergens in food manufacturing, it is impossible to reduce risk to zero.

Strongly agree ❑

Somewhat agree ❑

Somewhat disagree ❑

Strongly disagree. ❑

29. **[IF 'Parents' AT Q6 ASK]** How likely would you be to purchase a food product for your child in the following scenarios?

[IF 'Adults' AT Q6 ASK] How likely would you be to purchase a food product in the following scenarios?

**[RANDOMIZE]**	Definitely Would Not Purchase	Probably Would Not Purchase	Might or Might Not Purchase	Probably Would Purchase	Definitely Would Purchase
You could be assured that the small amount of the allergen, if present in the food, is not capable of triggering an allergic reaction	❑	❑	❑	❑	❑
You could be assured that the small amount of the allergen, if present in the food, is only capable of triggering a mild allergic reaction, such as tingly lips, an itchy throat or a mildly upset stomach	❑	❑	❑	❑	❑
You could be assured that the small amount of the allergen, if present in the food. is only capable of triggering a mild allergic reaction in 5% of people with that allergy, such as tingly lips, an itchy throat or a mildly upset stomach	❑	❑	❑	❑	❑

SECTION 3: Future state

[SHOW Q31a, b & c ON SAME SCREEN]

30. Currently, precautionary allergen labelling such as "may contain" or "manufactured in a facility that also processes…'your allergen'" statements are voluntary for food manufacturers and are not specifically regulated by Health Canada. Each manufacturer determines if and when a precautionary allergen label is needed based on their own manufacturing practices. Prior to starting this survey today, were you aware of this?

Yes	❑
No	❑

31. Food Allergy Canada is advocating to have greater guidance for the food industry on when precautionary allergen labelling should be used on pre-packaged food products. This would include using a scientific, risk-based approach to determine the need for precautionary allergen labelling based on how allergen risks are being managed throughout the manufacturing process. Pre-packaged products would undergo a risk assessment and a level of risk would be determined. Following the risk assessment, a manufacturer would then decide on the need for a precautionary allergen label. What are your reactions to this?

(Select ALL that apply)

[RANDOMIZED]

I thought or assumed this was already being done	❑
I agree that a standardized, scientific, risk-based approach to precautionary allergen labelling is needed	❑
It should be left up to food manufacturers to decide what precautionary labels to put on their products	❑
**[ALWAYS SHOW LAST**] Other (please specify)___________	❑

32. In your opinion, would a standardized, scientific, risk-based approach to precautionary allergen labelling as described above…

a) Make precautionary allergen labelling…

More confusing	❑
Equally confusing	❑
Less confusing	❑

b) Make precautionary allergen labelling…

More useful	❑
Equally useful	❑
Less useful	❑

c) Make you…

More confident purchasing food products	❑
Equally confident purchasing food products	❑
Less confident purchasing food products	❑

d) Make you..

More likely to purchase a food product with precautionary allergen labelling	❑
Equally as likely to purchase a food product with precautionary allergen labelling	❑
Less likely to purchase a food product with precautionary allergen labelling	❑

33a. When you see a "may contain" statement, what questions would you want answered by food manufacturers?

33b. How do you want to be communicated to by manufacturers regarding their allergen management practices? e.g., website, apps, social media, product packaging

SECTION 4: Basic Data

Finally, a few questions about you for data classification purposes.

34. **[ASK TO FAC DATABASE RESPONDENTS ONLY]** Did you watch Food Allergy Canada's webinar at the end of September 2021 that featured Dr. Philippe Bégin speaking about food allergen thresholds? The webinar was titled "Understand the severity of your food allergy".

Yes	❑
No	❑

35. **[IF 'Parent' AT Q6 ASK]** Has your child with the food allergy done an oral food challenge? And by oral challenge we mean a test performed in a medical facility under the supervision of an allergist to prove that someone is allergic to a particular food, or to show they are not allergic (or no longer allergic).

[IF 'Adult' AT Q6 ASK] Have you done an oral food challenge? And by oral challenge we mean a test performed in a medical facility under the supervision of an allergist to prove that someone is allergic to a particular food, or to show they are not allergic (or no longer allergic).

Yes, in the past 3 years	❒
Yes, more than 3 years ago	❒
No	❒

36. **[IF 'Parent' AT Q6 ASK]** Is your child current doing or have they ever done Oral Immunotherapy (OIT)? And by Oral Immunotherapy we mean treatment conducted under the supervision of an allergist, whereby small amounts of the food allergen are given in gradually increasing doses until the patient is able to eat a certain amount of it without having a reaction.

[IF 'Adult' AT Q6 ASK] Are you doing or have you ever done Oral Immunotherapy (OIT)? And by Oral Immunotherapy we mean treatment conducted under the supervision of an allergist, whereby small amounts of the food allergen are given in gradually increasing doses until the patient is able to eat a certain amount of it without having a reaction.

Yes	❒
No	❒

37. Do you identify as…

Male	❒
Female	❒
Non-binary / gender non-conforming	❒
Prefer not to answer	❒

38. Finally, which of the following categories best reflects your total annual household income before taxes?

<$25,000	❑
$25,000 to $49,999	❑
$50,000 to $74,999	❑
$75,000 to $99,999	❑
$100,000 to $149,999	❑
$150,000 to $199,999	❑
$200,000+	❑
Prefer not to answer	❑

Thank you for completing this survey. We greatly appreciate your feedback.

FOR FAC DATABASE RESPONDENTS: Route to Food Allergy Canada website

FOR PANEL RESPONDENTS: Route to panel
