# Supplementary material for: A Survey of Allergic Consumers and Allergists on Precautionary Allergen Labelling: Where Do We Go from Here?
Source: Nutrients. 2025 Apr 30;17(9):1556. doi: 10.3390/nu17091556 (PMC12073677; doi:10.3390/nu17091556)
Supplement: Supplementary file 1 [file nutrients-17-01556-s001.zip › Supplementary File S2.pdf]

ALLERGIST SURVEY QUESTIONS

A. Practice Information

1. In which Province/Territory do you currently practice?

- ☐ Alberta
- ☐ British Columbia
- ☐ Manitoba
- ☐ New Brunswick
- ☐ Newfoundland and Labrador
- ☐ Northwest Territories
- ☐ Nova Scotia
- ☐ Nunavut
- ☐ Ontario
- ☐ Prince Edward Island
- ☐ Quebec
- ☐ Saskatchewan
- ☐ Yukon

2. What is your patient population?

- ☐ Adult
- ☐ Pediatric (under 18 years)
- ☐ Both

3. How long have you been practicing?

- ☐ <1 year
- ☐ 1-5 years
- ☐ 6-10 years
- ☐ 11-20 years
- ☐ 20 years or more

4. In what clinical setting (s) do you currently practice? Select all that apply

- ☐ Academic hospital center
- ☐ Community hospital center
- ☐ Outpatient clinic (private or public)
- ☐ Other

5. **Approximately how often do you perform oral food challenges in your practice?**
   - ☐ Daily
   - ☐ Weekly
   - ☐ Once a month
   - ☐ Rarely (a few times a year)
   - ☐ Never

B. Precautionary Allergen Labelling

6. **Do you think PAL is currently a useful tool for patients to make informed decisions on the safety of pre-packaged foods?**
   - ☐ Yes
   - ☐ No
   - ☐ Unsure

Comment (open text box)

7. **Do you allow allergic patients to consume foods with PAL? Select all that apply.**
   - ☐ Adults
     - o Always
     - o Sometimes
     - o Never
     - o N/A

Please indicate your primary reasons for your response – open text box

   - ☐ Pediatric (under 18 years)
     - o Always
     - o Sometimes
     - o Never
     - o N/A

Please indicate your primary reasons for your response – open text box

8. **Does the type of food allergen listed in the PAL statement influence your recommendations to patients (e.g. Would you allow products with “May contain peanut” but not “May contain egg”)?**
   - ☐ Yes
   - ☐ No

If yes, for which allergens do you allow products with PAL (select all that apply)

   - ☐ Eggs

- ☐ Milk
- ☐ Mustard
- ☐ Peanuts
- ☐ Crustaceans and molluscs
- ☐ Fish
- ☐ Sesame
- ☐ Soy
- ☐ Tree Nuts (almonds, Brazil nuts, cashews, hazelnuts, macadamia nuts, pecans, pine nuts, pistachios and walnuts)
- ☐ Wheat

Insert Comment Box (open text box)

9. Are your recommendations to avoid foods with PAL influenced by skin prick test size / specific IgE levels?

- ☐ Yes
- ☐ No
- ☐ It depends – please specify:

Insert open comment box:

10. Are your recommendations to avoid foods with PAL influenced by an oral food challenge reactivity threshold?

- ☐ Yes
- ☐ No
- ☐ I do not perform oral food challenges.

Insert open comment box:

11. Do you use a specific cut-off value (e.g. in mg of protein)?

- ☐ Yes
- ☐ No

If yes, please indicate the cut-off value used – if applicable, specify if different cut-offs are used for different allergens- open text box.

C. Allergen Thresholds

While some food-allergic patients react after exposure to small quantities of allergen, it is now well-established that food-allergic patients have individual threshold doses below which they will not experience an adverse reaction.

As there has now been more progress on understanding individual thresholds, if industry applied PAL based on a risk assessment using a set population threshold, it has the potential to provide more clarity of what the PAL actually means, thus reinforcing it as a useful tool for those with food allergy. It also has the potential to allow some allergic individuals to consume prepackaged foods with PAL **IF,** for that individual, their threshold is above the stated amount in the PAL. In this context, a single-dose oral food challenge could be offered with a quantity corresponding to this defined threshold dose or slightly above to assess tolerance to foods with PAL.

The following questions are intended to get your input on this use of thresholds to inform PAL and how this might translate into clinical practice.

12. Would you agree with the fact that PAL labelling <u>should not be used</u> when the presence of a contaminating allergen is unlikely to trigger an allergic reaction in the vast majority of patients?

- ☐ Yes
- ☐ No
- ☐ Unsure

Comments: (open text box)

13. Would you be prepared to perform a single-dose oral food challenge to assess the tolerance to a small defined quantity of an allergen for the purpose of determining whether or not your patient can consume certain foods with PAL?

- ☐ Yes
- ☐ No
- ☐ Unsure

Comments: (open text box)

14. What challenges do you foresee in providing guidance on PAL products to your patients using the approach outlined in the question above? Select all that apply.

- ☐ Lack of patients' understanding of allergen thresholds
- ☐ Patients' hesitancy in consuming a product that contains their allergen, even at the lowest level

- ☐ Limited access to oral food challenges for patients
- ☐ Challenges in performing a single-dose food challenge (ex: which type of food to use, measuring the appropriate quantity of food, office/clinic capacity and staffing etc.)
- ☐ Understanding that thresholds may sometimes change under certain circumstances (infection, exercise etc.)
- ☐ Challenges for patients with multiple food allergies
- ☐ I do not foresee any challenges.
- ☐ I will not perform single-dose challenges

Comments: (Open comment box)

15. Please provide any additional comments – open text box.
