# Supplementary material for: A Survey of Allergic Consumers and Allergists on Precautionary Allergen Labelling: Where Do We Go from Here?
Source: Nutrients. 2025 Apr 30;17(9):1556. doi: 10.3390/nu17091556 (PMC12073677; doi:10.3390/nu17091556)
Supplement: Supplementary file 1 [file nutrients-17-01556-s001.zip › Supplementary Tables.pdf]

Table S1. Factors taken into account when allowing patients to introduce foods with precautionary allergen labelling (PAL)

Reliability of family using epinephrine
Severity of index reaction.
Comorbidities (asthma…).
Reaction threshold.
Previous tolerance of foods with PAL (ex: pursue same brand if tolerated in the past).
Anxiety of patients.
Type of company ("well-established company").
Origin of products (Canadian vs International).
Risk-benefit/shared decision making/risk tolerance discussion
Tolerance of baked forms of allergen (baked milk/egg).
Leave it up to patients

Table S2. Challenges foreseen by allergists in providing guidance on precautionary allergen labelled products based on a single-dose oral food challenge.

Type of challenge foreseen	Number of responders (%)
Thresholds may sometimes change under certain circumstances	44 (83)
Limited access to oral food challenges for patients	36 (67.9)
Lack of patients' understanding of allergen thresholds	31 (58.5)
Challenges for patients with multiple food allergies	31 (58.5)
Patient's hesitancy in consuming a product that contains their allergen, even at the lowest level	30 (56.6)
Challenges in performing a single-dose food challenge	29 (54.7)
I do not foresee any challenges	0 (0)
